# Peroneus Tenotomy

**Published:** 1898-08

**Authors:** 


PERONEUS TENOTOMY. Peroneus tenotomy, together with division of the tibial fascia, seldom fails to relieve the worst cases of stringhalt.

